# Grand (meta) challenges in planetary health: environmental, social, and cognitive

**DOI:** 10.3389/fpubh.2024.1373787

**Published:** 2024-12-05

**Authors:** Colin David Butler

**Affiliations:** ^1^National Centre for Epidemiology and Population Health, Australian National University, Canberra, ACT, Australia; ^2^Institute for Climate, Energy and Disaster Solutions, Australian National University, Canberra, ACT, Australia

**Keywords:** novel entities, climate change, civilization collapse, limits to growth, optimism bias, population bias, scientific integrity

## Abstract

The term “planetary health”, coined in the 1970s, arose from planetary consciousness, stimulated in part by the dawn of the space age, and commensurate recognition that our species faces extraordinary obstacles (“limits to growth”) if it is to fulfil its promise. While such awareness was then widely suppressed, awareness is reviving, driven by the now obvious perils, not only of climate change but also from weaponization and national aggression. Our neoliberal society (including in academic circles) has inappropriately rewarded articles and researchers that are biased toward optimism. This article proposes six grand (“meta”) challenges that planetary health must face.

## Introduction

Planetary health is an exciting idea. Over 3,200 other known stars are orbited by planets; many await discovery (1). Even if life proves common, our civilization, created by primates with a complicated family tree (2), is precious. Its durability requires stewardship (3, 4). Yet, it is at risk, particularly from conflict, perhaps worsened by artificial “intelligence”, including “Lethal Autonomous Weapon Systems” (5).

Civilization evolved during the Holocene—the current interglacial, following a much longer Ice Age. Until recently, this was regarded as destined to soon terminate (driven by orbital changes), but increased energy in the Earth system, trapped by rising greenhouse gases, may now postpone this for scores of millennia (6).

Technology has transformed our planet during the Anthropocene, the human-dominated era (4). While this has benefitted most humans (though few other species), some change has been accidental, such as the initial accumulation of greenhouse gases (GHGs). Note that the scale of current GHG emissions can no longer be considered inadvertent. Until recently, it was believed that pollution due to human activities could cause only local harm but it is now understood that the linked Earth–human system has many limits and thresholds, vulnerable to human pollution and other actions (7).

## Early recognition of links between human and planetary health

Aspects of Earth system “health” (including nuclear war) have been understood by health workers as relevant to human health for over six decades (8). During the space race, global consciousness that humans share one small planet stimulated seminal books (9). *Silent Spring* (10) warned of the bioaccumulation of synthetic chemicals—biocides—later termed “novel entities” (11) and their risk to ecological and human health. Another was by Dubos, a leading microbiologist (12), who, in the 1960s, developed into a prize-winning biosphere activist and co-authored a book subtitled *The Care and Maintenance of a Small Planet* (13).

In the 1970s, human ecologists published in health journals (14, 15); one (Sargent) warned of “manipulations of the processes of the planetary life support system”. Seminal was *The Limits to Growth*, released in 1972 (16). Selling over 12 million copies in 30 languages, its authors used computer modeling to explore interactions among population growth, resource demand, industrialization, food production, and pollution. This study warned that the continuation of existing trends (i.e., that deny the existence of limits) would lead to a marked decline in human wellbeing, including total population size, within a century (9).

In the 1980s, Hansen, a particularly skilled scientific communicator, repeatedly testified to the US Senate concerning climate change (17). His activism is credited as stimulating the first widespread public awakening to this risk.

Leading health journals also paid attention; in 1989, *Lancet* published an inaugural editorial on climate change. However, in 1989, Hansen began to complain of government suppression (17).

## Early uses of the term “planetary health”

Prescott et al. have traced the term planetary health to the 1970s, to the “interdependence between human health and place at all scales” (18). They noted that activists called (in 1980) for an expansion of the World Health Organization's (WHO) definition of health to acknowledge that it “involves planetary health”. In 1990, King called for incorporating “sustainable” into the same definition (19). He referred to “the health of the planet” and noted the “contribution to planetary ill health” made by the industrialized world. In 1991, Lovelock, best known for the “Gaia” hypothesis, published a book subtitled *The Practical Science of Planetary Medicine* (20). In 1994, epidemiologist McMichael published *Planetary Overload* (21) building, in part, on Sargent's warning about exceeding “life support mechanisms” (15). A 1998 report signified growing WHO recognition of these concepts (22).

In 2014, a “manifesto” of planetary health was published (23), followed by a detailed report (24). Each explicitly warns of “threats to the sustainability of our civilization”, while the report reminds readers that “human health and human civilization depend on flourishing natural systems” and their wise stewardship.

## Six grand (meta) challenges in planetary health

I next sketch six selected meta-challenges for planetary health. The meaning of “meta”, from the Greek, is “to go beyond”, such as in “metaphysics”, the study of nature at a deeper level. This list is, of course, based on my judgment; it is not intended to be didactic, comprehensive, or exclusive. However, I believe each is very important. These challenges also have relevance beyond planetary health. I call for greater transparency, ethical behavior, and courage to consider the unthinkable—to stimulate policies to avoid civilization's collapse (25).

### Reduce self-censorship, challenge power

We need greater scientific courage. Although Earth system indicators are abundant, their interpretation is uncertain and debated, primarily as they involve the future (26). There is growing evidence that Earth system and health scientists have erred toward optimism.

In 2005, soon after my experience with the future scenarios section of the Millennium Ecosystem Assessment (MEA), I published an essay arguing that thresholds are insufficiently factored into models of future population size, leading to absurd projections of maximum human population (26). Hansen has suggested that ‘scientific reticence' has delayed public recognition of climate change's risk (27), and others (28) argued that scientists generally err toward reassurance. Only the most secure scientists have been able, consistently, to call for truly fundamental changes in awareness and behavior.

**Figure 1:**
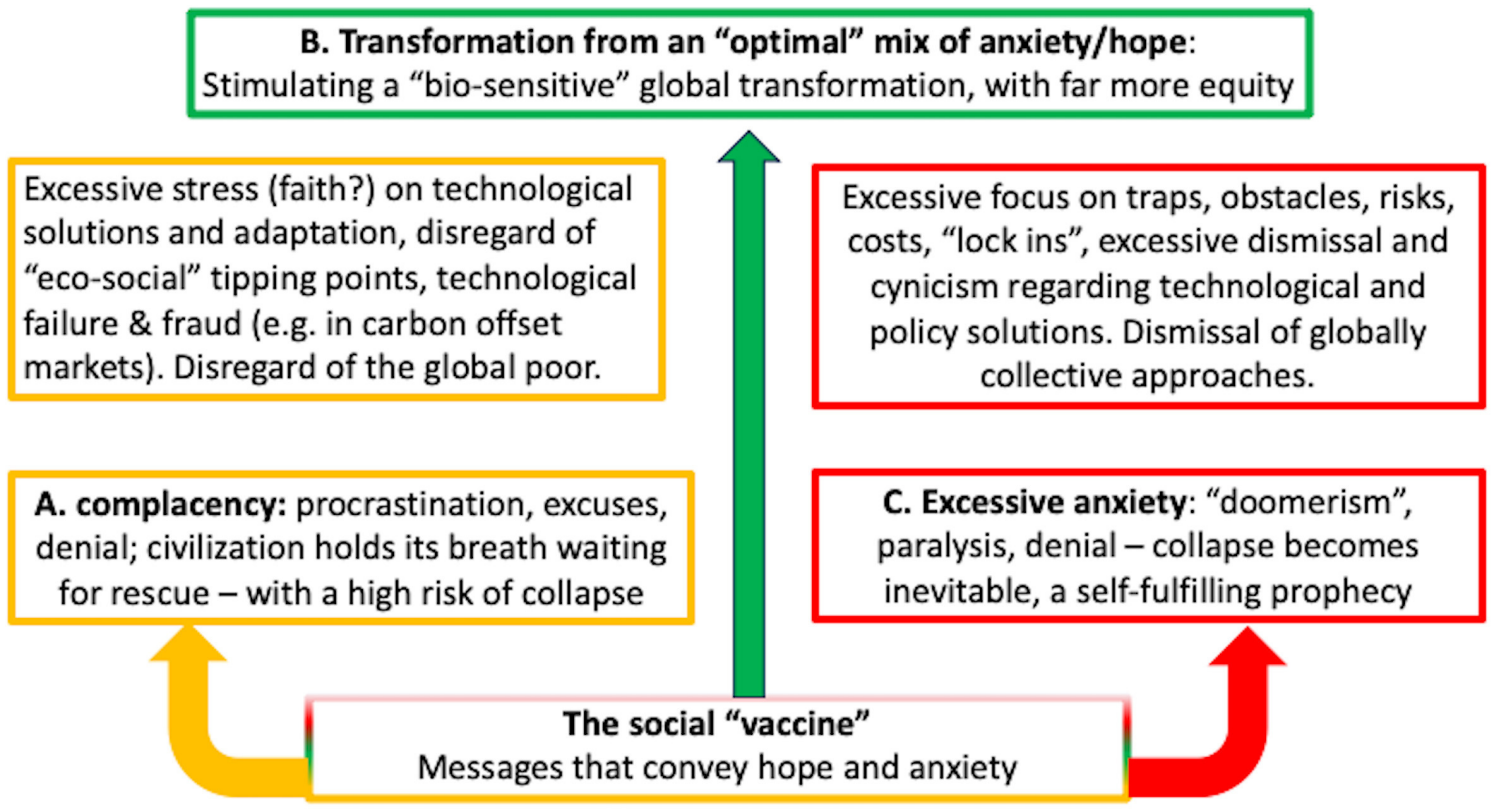
Excessive “dose” of either optimism or pessimism is harmful. Adapted from Butler et al. (56).

Consequently, substantial parts of the Earth system literature are biased toward optimism (Figure 1), including “negative emission technologies”, claiming to rescue us in the near future from catastrophic warming (29, 30). A waltz between money-allocating policymakers (whose loyalty remains overwhelmingly to fossil-fuel companies) and grant-seeking scientists has left future society enormously vulnerable to the failure of unproven strategies. Senior scientific figures who have recognized this include a former director of the Intergovernmental Panel on Climate Change (30). Hansen is also an exception; thus, his forecast that the deliberate pollution of the atmosphere with sulfate aerosols may soon be required to attempt to reduce global heating is disturbing (31).

Supporters of the “precautionary principle” argue for wide safety margins. In many fields, this is uncontroversial: Most nation states have military forces to deter invasion; individuals with means purchase insurance; farmers delay planting until the right season. However, for decades, excuses have been found to avoid and to seek to defer costs and changes necessary to secure planetary health.

This colossal failure has many explanations. One is that the emergency, soon after its recognition, was forecast as maturing in what then seemed the far future—the mid-21st century. A second reason is the vehement, skilled organization of corporation-led opposition, allied with political power, to any attempt to restrain or redefine economic “growth” (32). It has been argued that “climate change is not the result of a market failure but rather the outcome of a fully functioning capital accumulating economy working hard to shift costs on to others” (29).

A third reason is a human genetic and cultural bias toward optimism (33). In addition, humans have limited experience with rapid global environmental change (34). During the transition to the Holocene, sea levels rose by 60 meters over several millennia (35). However, the human population size was then much smaller, and resources were more abundant. Our ancestors prospered in the warmer climate. Warmth is good, but like many natural phenomena (e.g., serum potassium levels), the issue is dose.

### Regard nature as an ally, not a slave or a foe; recover humility

Humans have had incredible success at transforming nature. Catton has argued that this has led to hubris and “overshoot” (36). Piguet (37) has analyzed reasons for the recent disappearance of “environmental considerations” to explain human displacement (now at a record high); he quotes Glacken: “the history of mankind is the history of the conquest of nature” and Beck: a modern society “increasingly develops outside nature.” Such pronouncements are naïve.

A paper on the risk to the Mekong delta of inundation via subsidence and sea-level rise calls for revising the “strong belief in human mastery over nature”. This delta currently produces 7–10% of all rice traded internationally (38). Its potential flooding is but one of the innumerable problems that we face.

### Restore trust in science—Including publishers

Trust in science is vital to winning public support for the radical changes needed to ensure planetary health. Yet, this is falling, including from phenomenal rates of fraud in the scientific literature (39).

Fake research “threatens to overwhelm the editorial processes of a significant number of journals” (39). Another consequence is confusion among early-career researchers and journalists. Separating published chaff from passable articles is not always easy. How can novices determine flaws in the pyramid of evidence upon which new science sits if many “peer-reviewed” articles are fabricated?

The country of origin of retracted papers has been reported as five times more from the People's Republic of China than from the US (40). A driver for this fraud is excessive reliance for promotion on decisions by the Web of Science, a database controlled by a private corporation (Clarivate) whose processes involved in calculating publishing metrics are criticized as “unscientific and arbitrary” (41). Over-reliance on metrics to assess merit is highly problematic. Loss of confidence in science is further amplified by “mega” (42) and “predatory” publishers.

### Foster nuanced, mutually respectful discussions about population and consumption

The issues of population (including growth rates and absolute numbers) and resource consumption have long been understood as integral to planetary health. However, many misconceptions remain, including some propagated in a recent major report of the UN Population Fund (UNFPA) (43). In this, UNFPA's executive director asserted that its second key message is to “shatter the myth” that experts “blame fertility rates for the climate crisis” (44). To the contrary, experts overwhelmingly attribute climate change mostly to the behavior of populations in high-income settings, including via their purchase of products from the global south. Most scholarly concern about high population growth rates in “developing” countries focuses on consequences for poverty, hunger, vulnerability to climate change, and other aspects of impaired planetary health (45).

### Clearer discussion of links between conflict, displacement, and planetary health

**Figure 2:**
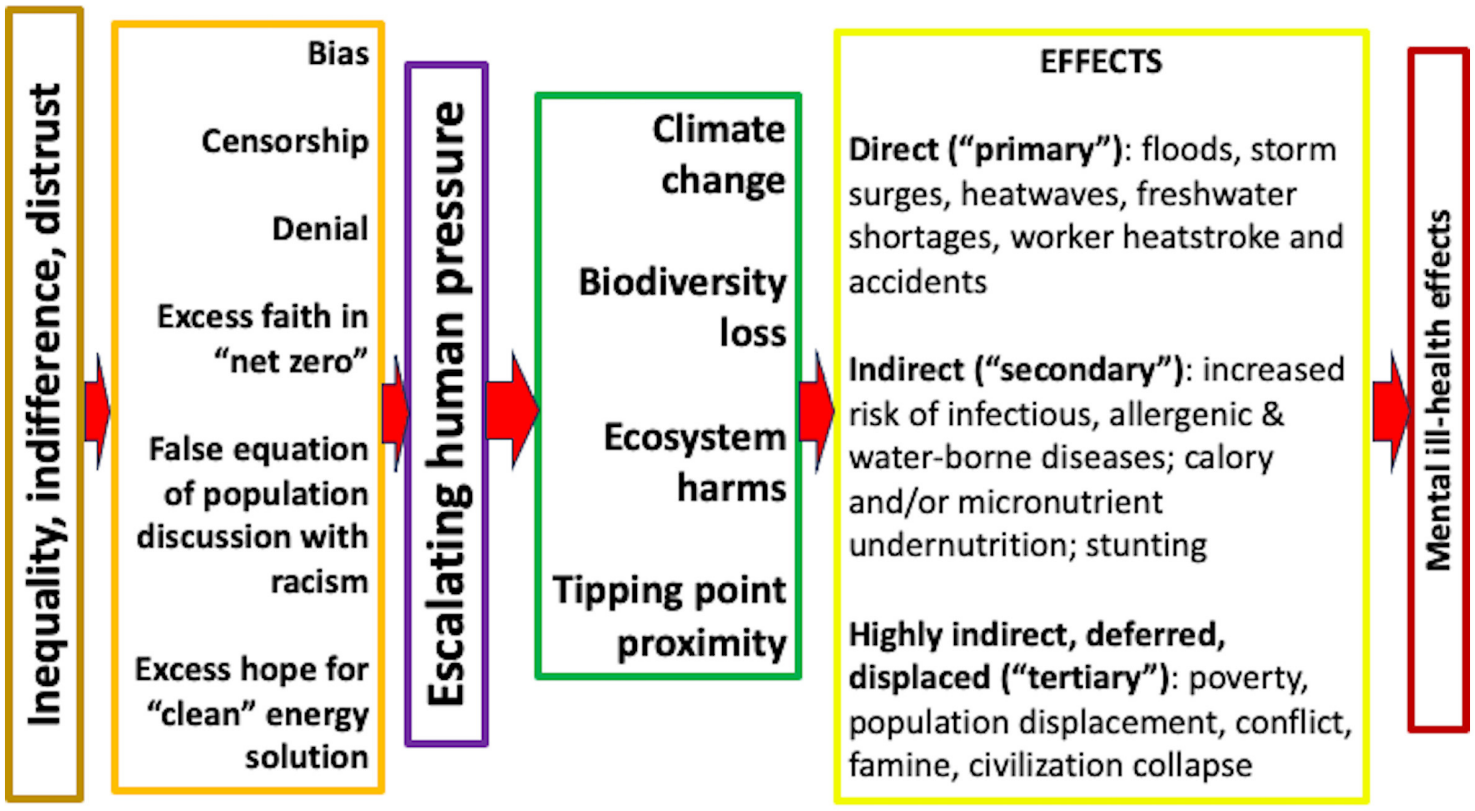
Mechanisms by which the harmful effects of ecosystem change can affect human health. Adapted from Corvalan et al. (57) with permission from WHO, which is not responsible for the content or accuracy of this adaptation.

Neither paper that revived the term planetary health (23, 24) has much discussion of conflict or population displacement. However, one (24) reproduces a figure that groups health effects into three classes (Figure 2). The third category includes conflict. This typology has the advantage of identifying a hierarchy of effects among the thousands of adverse health consequences of failing planetary health (46).

Recent planetary health papers acknowledge debts to the literature on planetary and Earth system boundaries (47) and thus to the Limits to Growth (48). However, as yet, there has been little conceptual development of the risk to health from conflict or population displacement as regional and global limits are approached or breached (49). A likely reason for this self-censorship is fear of disciplinary transgression; another may be fear of causing excess concern. If so, that is unwarranted—risks need accurate forecasts to be reduced (25).

### Stronger regulation of synthetic biology and bioweapon capacity

Synthetic biology includes the genetic modification of organisms and viruses. Modern capacity for this extends far beyond the selective breeding of plants (and gene insertion in laboratories), seeking to enhance global food security. It also includes the manipulation—and even the creation—of known and novel pathogens, some of which might cause pandemics. The risk from such “biohazards” was recognized in 1975 at the first Asilomar Conference (50). A summary statement from this meeting agreed to confine some experiments to highly secure laboratories to reduce risk. Since then, however, the power of synthetic biology has increased immeasurably, and many pathogen leaks have occurred from supposedly secure laboratories (51).

In 2018, researchers reported the synthesis of horsepox from chemically synthesized DNA fragments. They explained they did this to show that synthesis of variola (which causes smallpox) is now possible (52). The creation of novel pathogens is relevant to planetary health not only because COVID-19 has shown how harmful pandemics can be to global wellbeing but also because such pathogens, if previously unknown in nature, are novel entities, one of the planetary boundaries (53). The origin of SARS-CoV-2 remains unknown (54, 55); continued downplaying its possible laboratory origin also undermines public trust in science.

## Conclusion

The planetary health emergency is deepening. Excellent research can inform policymakers on ways to reduce our common peril.

## Data Availability

The original contributions presented in the study are included in the article/supplementary material, further inquiries can be directed to the corresponding author.
